# Aerosol Dispersion and Efficacy of Protective Strategies During Dental Procedures

**DOI:** 10.1016/j.identj.2025.01.015

**Published:** 2025-02-12

**Authors:** Mustafa Radif, Andrew Young, Eric Salmon, David M. Ojcius, Shika Gupta

**Affiliations:** Arthur Dugoni School of Dentistry, University of the Pacific, San Francisco, CA, USA

**Keywords:** Aerosols, Oral health, Dentistry, Infectious diseases

## Abstract

**Introduction and Aims:**

Aerosol generation during dental procedures poses significant risks due to the potential for transmitting aerosol-bound microorganisms, including those in dental unit waterlines. This study aimed to quantify aerosol dispersion at various distances from dental procedures using a high-speed electric handpiece, with a focus on the effectiveness of various aerosol mitigation strategies.

**Methods:**

Employing a mannequin head with an artificial tooth (typodont), we simulated clinical settings without the use of saliva to solely assess the contribution of dental unit waterlines and mechanical factors to aerosol production. Measurements were taken using a spectrometer at distances of 0, 0.9, and 1.8 meters from the handpiece.

**Results:**

The results showed no significant difference in aerosol dispersion between 0.9 and 1.8 meters without evacuation. In contrast, the use of high-volume evacuators, particularly the Isolite system, significantly decreased aerosol dispersion across all distances.

**Conclusion:**

We found that any type of high-volume evacuator can decrease aerosol dispersion, but the use of Isolite was the most effective.

**Clinical Relevance:**

The results from this study can influence choice of safety measures to minimize aerosol spread during dental procedures.

## Introduction

Dental procedures are a significant source of aerosols, which are fine particles suspended in the air that can carry saliva, blood, microorganisms, and other contaminants.^1^^,^^2^ These aerosols are generated during various dental procedures (aerosol-generating procedures) such as those that use high-speed handpieces, ultrasonic scalers, and air-water syringes. The nature of these aerosols, compounded by the complexity of their sources, including not only biological fluids but also substances from dental unit waterlines (DUWLs), necessitates a comprehensive understanding of their composition, sources of generation, associated risks, and the effect of mitigation strategies to ensure safety within dental settings.

The term ‘dental aerosol’ refers to the suspension of particles generated during dental interventions. These aerosols consist primarily of saliva droplets and irrigant that can contain pathogens such as bacteria or viruses present within the oral cavity as well as blood particles from gingival bleeding.^3^ The high-speed handpieces used in dentistry create a significant amount of turbulent airflow, which propels these saliva droplets into smaller sizes that remain airborne for extended periods,^3^ increasing the risk of inhalation and subsequent infection.

Furthermore, ultrasonic scalers produce intense vibrations that atomize water coolant into aerosols laden with biofilm debris, tooth debris, and dental materials,^4^ adding another layer of risk for contamination.

Exposure to dental aerosols poses multiple hazards for patients and healthcare professionals as inhalation or direct contact with contaminated droplets can lead to respiratory tract infections caused by pathogenic microorganisms present within the aerosols.^5^^,^^6^ The advent of severe acute respiratory syndrome coronavirus 2, responsible for the coronavirus disease of 2019, has dramatically highlighted the critical importance of understanding and mitigating the risks associated with dental aerosols.^7^, ^8^, ^9^, ^10^ Recent research has demonstrated that severe acute respiratory syndrome coronavirus 2 can be transmitted through aerosols generated during certain dental procedures.^5^ Furthermore, DUWLs are another potential source of pathogens, such as *Legionella pneumophila,*^11^^,^^12^ which could be carried by aerosols. This underscores the need for infection control measures to prevent disease transmission.

To effectively minimize the risks posed by dental aerosols, a variety of strategies can be employed in clinical settings. The use high-volume evacuators or suction devices during procedures to capture and remove aerosols at their source, significantly reducing the risk of aerosol dispersion.^13^ Preprocedural mouth rinses containing antimicrobial agents have been shown to significantly reduce the bacterial load in saliva, mitigating potential contamination.^14^ Moreover, the implementation of air purification systems equipped with HEPA filters can effectively remove airborne contaminants from the dental environment.^13^ The design of handpieces (air turbine vs electric handpiece) and DUWL disinfectants are also important bioaerosol control measures.^15^^,^^16^ Strategic use of dental rubber dams during procedures further helps in minimizing the generation and spread of aerosols, effectively isolating the treatment area from the oral environment.

The objectives of this study are to determine the concentration of aerosol particles at various distances from a dental procedure when using a high-speed electric handpiece without tooth preparation or the use of an evacuation system and to evaluate the efficacy of different evacuation systems in reducing these concentrations during simulated tooth preparation with high-speed electric handpieces.

## Materials and methods

**Fig. 1:**
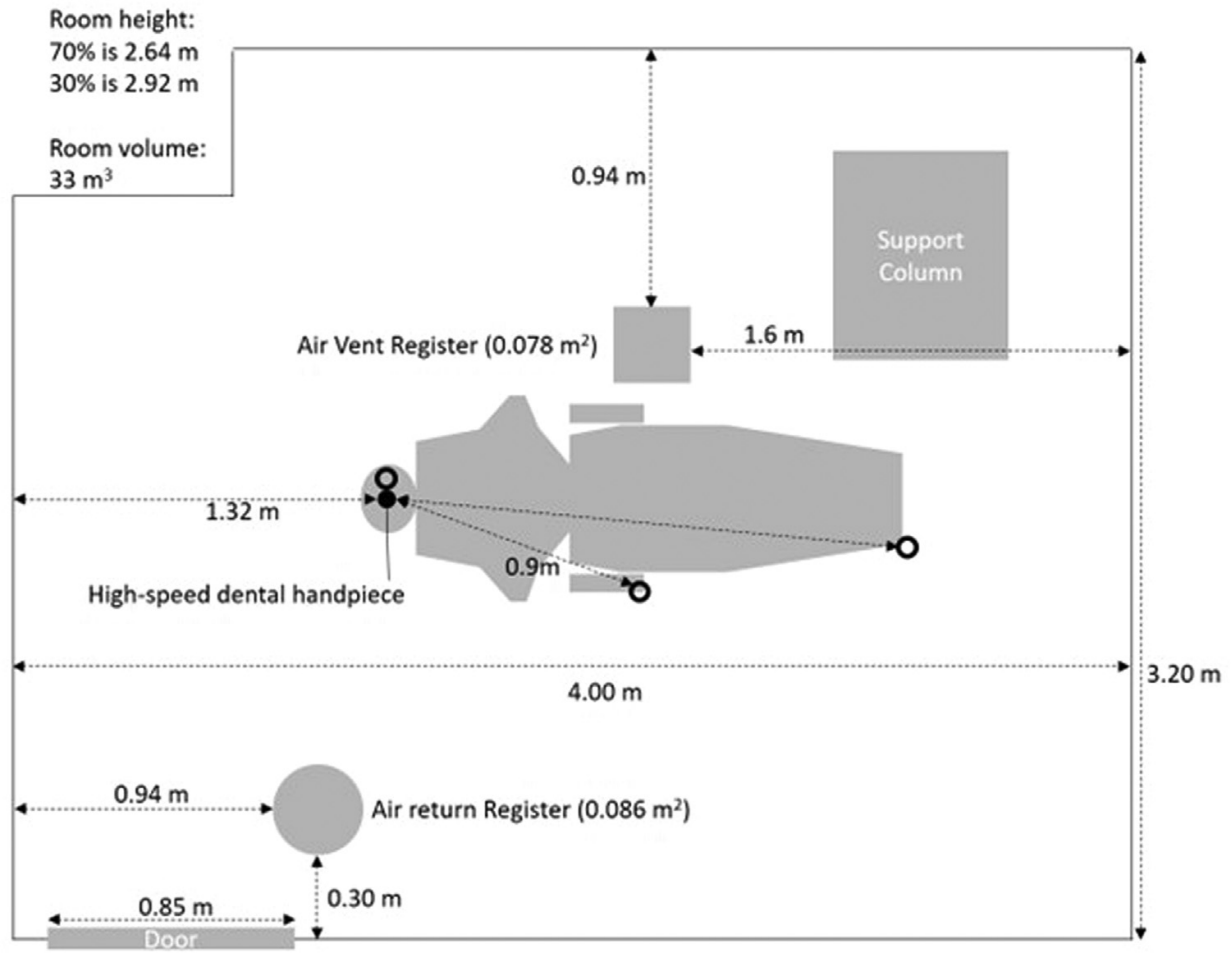
**Room measurements.** The door remained closed during all measurements. O = site of particle measurements.

The trials were conducted in a closed-room clinical operatory at the University of the Pacific, School of Dentistry, San Francisco, California, USA. The operatory was not utilized for any procedures for 24 hours prior to the trials. The room was rectangular in shape with dimensions of roughly 4 m long by 3.2 m wide by 2.6 m high (Figure 1), resulting in a volume of roughly 33 cubic meters, with 6 to 8.5 air changes per hour. No windows or doors were open during the experiments and the temperature was kept constant at 72 degrees Fahrenheit.

A mannequin head was mounted to the dental chair, which was reclined to the supine position and standardized for all trials. The mannequin head had artificial jaws and teeth (Frasaco, GmbH). A right-handed operator sat at the 11 o'clock position and performed all the trials. The operator wore a surgical gown, surgical mask, eye protection, hair covering, and shoe coverings to avoid breathing the aerosols and protect clothes and shoes.

We used the 3330 Optical Particle Sizer (OPS) (TSI, which provides count, size distributions, concentration, and total count for particles with optical diameters from 0.3 to 10 μm). Particle pulses are sized and binned in up to 16 different channels. For this study, all 16 channels were used to collect data. The aerosol concentration range for the OPS is 0 to 3000 particles/cm^3^. The concentration measured by the OPS is sensitive to the flow rate and hence the flow was tightly controlled to 1.0 L/min ±5%. The spectrometer was connected to a computer with Aerosol Instrument Manager software installed to provide computer-controlled operation, data collection/data interpretation, data importing/exporting, and printing.

We divided our research into 2 phases. For phase 1, the goal was to determine the number of aerosol particles at different distances using a high-speed electric handpiece, simulating tooth preparation and withholding evacuation systems. Prior to each trial, the 3330 OPS spectrometer collected ambient distribution of particles for 10 minutes during which time no aerosols were generated. The spectrometer collected data at 5-second intervals, hence 120 samples were collected over a course of 10 minutes for each preoperative measurement. A high-speed electric handpiece Kavo GENTLEpower LUX 25 LPR high-speed handpiece which creates a mist of coolant was run at 200,000 revolutions per minute, simulating tooth preparation. A 330 carbide bur was attached to the handpiece for simulating tooth preparation. The handpiece was run at 1 cm from the occlusal surface of lower left mandibular first molar for 7 minutes in a static position and without the use of any evacuation systems. The lower left mandibular first molar was chosen as studies have shown that it is most prone to caries even before it is fully erupted, and its anatomy makes it more prone to biofilm retention. In their study, Nader Nadershahi et al previously found that the number of aerosol particles generated by a high-speed rotary handpiece reached a plateau at 7 minutes (unpublished; personal communication).

The 3330 OPS spectrometer was placed at 0, 3, and 6 ft (0, 0.9, and 1.8 m) in front of the high-speed dental handpiece to measure aerosol concentrations (Figure 1). Six feet is the standard maximum distance used by the US Centers for Disease Control for determining whether an exposure has occurred. Three feet is half of that standard distance. The OPS spectrometer was placed 0.9 m above the floor for each of these distances. The spectrometer collected 84 samples for every 7-minute trial. Thirty minutes of fallow time was allowed between each trial. All trials for Phase 1 were carried out on the same day.

For phase 2, the purpose was to determine the efficacy of 3 commonly used evacuation systems in dentistry using a high-speed electric handpiece and simulating tooth preparation. As in phase 1, the 3330 OPS spectrometer collected ambient distribution of particles for 10 minutes prior to each trial, during which time no aerosols were generated. The evacuation systems used were: High volume evacuator (HVE), Rubber Dam with HVE, and Isolite (Zyris). The presence of a rubber dam can affect the flow dynamics of air and aerosols. The HVE was taped to the mannequin at 2 mm from the buccal surface of lower left mandibular first molar to standardize the position during the trials and approximates the typical position of the HVE by a dental assistant during dental procedures. The Isolite is a flexible attachment that, when placed in the mouth, helps with suction, retracts the tongue, and occludes the oropharynx.

Phase 1 trials were carried out once for each distance, and Phase 2 trials were carried out once for each distance and evacuation method. All phase 2 trials were conducted on the same day.

### Data analysis

The smallest four particle sizes (0.337, 0.419, 0.522, and 0.650 μm) were determined to be the most clinically relevant, and those samples were aggregated to calculate means, distributions, and standard deviations. Studies have shown that the exhaled cough aerosols from patients with respiratory infections are predominantly small particles.

All numbers and charts show the aggregated sum or mean of these 4 particle sizes. The absolute number of particles captured drops off by approximately 10-fold from 0.337 to 0.650 μm; therefore, the relative contribution of larger particles becomes increasingly small and inconsequential to the statistical analysis. The increase in operative aerosol levels over preoperative levels was calculated by subtracting each operative sample from the mean preoperative aerosol level in each scenario. Descriptive statistics for the increase in aerosols are presented in Table 1. Negative mean values are explained by an expected variation in ambient baseline aerosol levels.

**Table 1:** Mean particle concentration at various distances with various methods of aerosol mitigation.

		Distance from high-speed dental handpiece		
		0 m	0.9 m (3 ft)	1.8 m (6 ft)
Method of aerosol mitigation	No evacuation	591.4 (762.8)	21.5 (12.6)	20.7 (6.4)
	High-volume evaluation	108.1 (110.2)	2.5 (7.9)	26.1 (13.2)
	Rubber dam and high-volume evacuation	10.5 (6.5)	–15.7 (4.9)	3.7 (4.0)
	Isolite	–16.2 (5.4)	9.1 (4.7)	–6.5 (3.4)

Mean concentrations of 0.337 μm particles, over a period of 7 minutes, were measured at various distances from the high-speed dental handpiece, using various methods of aerosol mitigation. Concentrations are presented in dW/dDp (s.d.).

## Results

**Fig. 2:**
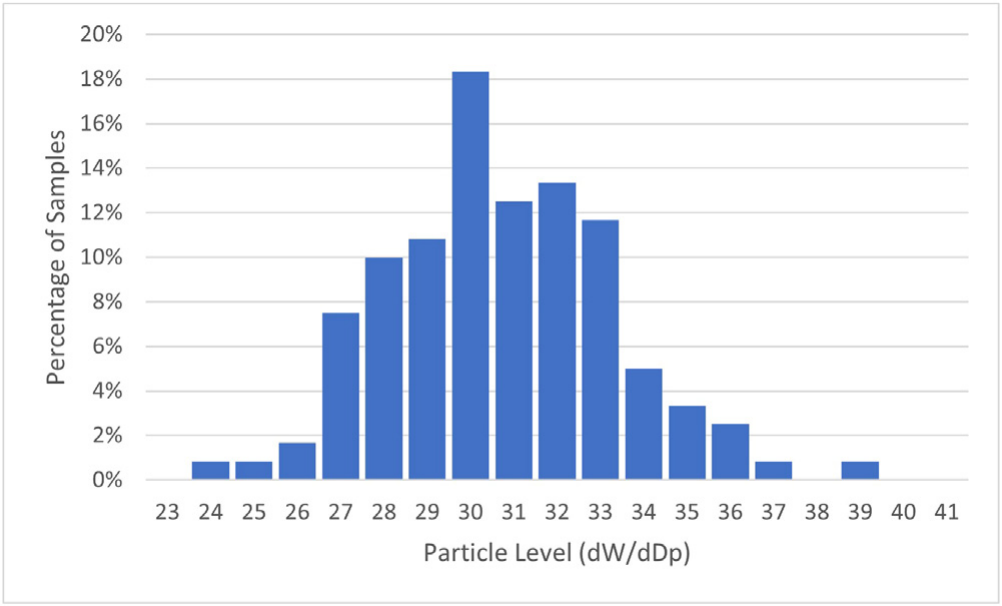
**Histogram** of representative baseline data sample at 0.337 µm particle size with 120 samples.

The distributions of aerosol level increase were determined by creating histograms for each particle size and distance. A representative distribution of the data at one particle's size can be seen in Figure 2. The small particle sizes produced normal distributions for postoperative and preoperative test runs, the exception being the larger particle sizes. With larger particle sizes and greater distances, the normal distribution became truncated due to an increasing number of samples showing no particles. Because of this, parametric tests requiring normal distributions were utilized during the data analysis. When distributions were compared pair-wise, such as when comparing pre-op particle levels to op particle levels or high-volume evacuation with and without a rubber dam, an independent t-test was used. When there were 3 or more groups, an ANOVA was used to analyse mean differences. The Levene Statistic always had the significance of <0.001 showing that variances were not homogenous and prompting the use of Games-Howell for post-hoc testing in every case.

**Fig. 3:**
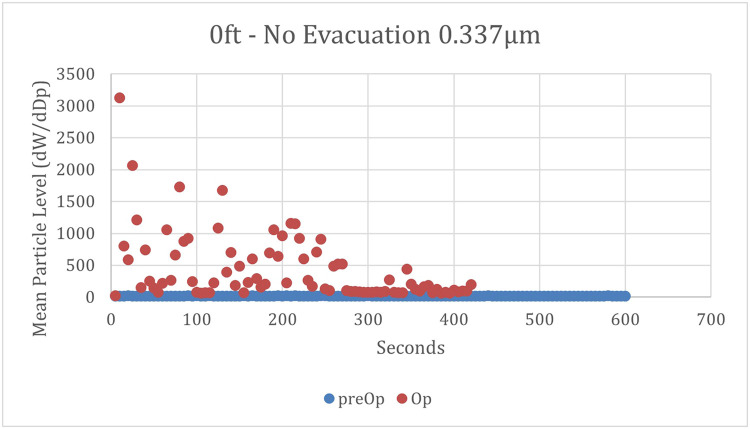
Mean particle concentrations over time at 0 meters and no evacuation. Mean concentrations of 0.337 µm particles were measured at the site of the high-speed dental handpiece, with no utilization of evacuation. ● = preoperative mean particle concentrations; ● = operative mean particle concentrations.

**Fig. 4:**
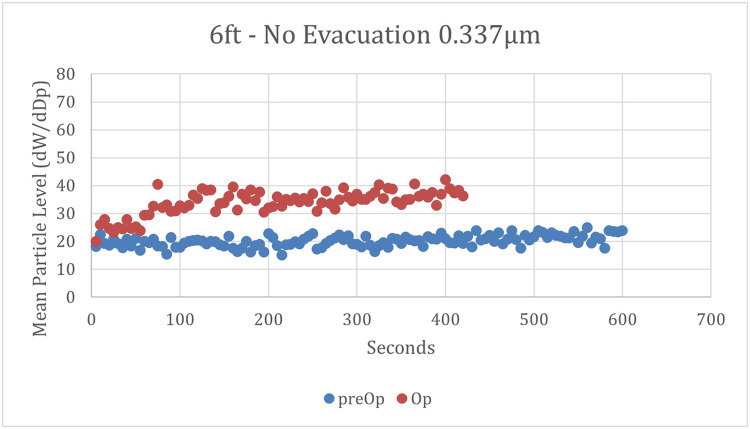
**Mean particle concentrations over time at 1.8 meters and no evacuation.** Mean concentrations of 0.337 µm particles were measured 1.8 m (6 ft) from the site of the high-speed dental handpiece, with no utilization of evacuation. ● = preoperative mean particle concentrations; ● = operative mean particle concentrations.

**Fig. 5:**
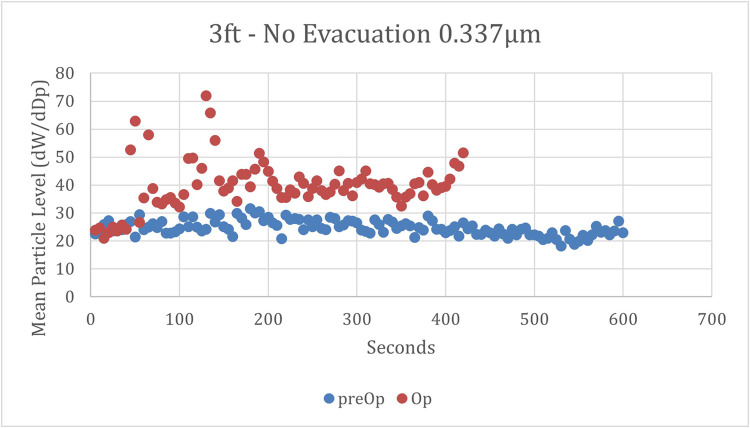
**Mean particle concentrations over time at 0.9 meters and no evacuation**. Mean concentrations of 0.337 µm particles were measured 0.9 m (3 ft) from the site of the high-speed dental handpiece, with no utilization of evacuation. ● = preoperative mean particle concentrations; ● = operative mean particle concentrations.

Without any evacuation at 0 meters, there was a large initial surge of particles, approximately 10 to 25 times higher than the highest measurements at 0.9 or 1.8 meters (3 or 6 feet) (Figure 3). This surge tapered off over time, stabilizing at a level significantly higher than the preoperative levels. At 1.8 m, there was a gradual increase from baseline during the first 2 minutes, as depicted in Figure 4. Levels then plateaued at 178% of baseline levels for the remainder of the testing cycle. At 0.9 meters, the results mirrored a combination of these patterns: an initial surge followed by a gradual increase from baseline, which then plateaued at 168% of baseline levels (Figure 5).

With the implementation of high-volume evacuation, no initial surge was observed at any distance. At both 0 and 1.8 meters, particle levels gradually increased, then plateaued; however, at 0.9 meters, particle levels remained barely distinguishable from baseline levels. Adding a rubber dam to the high-volume evacuation system resulted in similar patterns, but the increase was much smaller, barely above the baseline. Utilizing an Isolite system, particle levels maintained a stable level at or near the preoperative level.

Without evacuation at 0 meters, the highest mean increase in aerosol levels and the greatest variance were observed. Employing high-speed evacuation with a rubber dam consistently resulted in a decrease in mean aerosol quantities compared to conditions without a rubber dam. Across each tested distance, high-speed evacuation was compared to high-speed evacuation without a rubber dam. In all instances, the results were significant, with a *P* value <.001, indicating lower mean particle levels when a rubber dam was used. This suggests that utilizing a rubber dam effectively decreases particle levels across all studied distances when combined with high-speed evacuation. These results are consistent with a previous study, which found that a high-volume extraction device may lower the risk of transmission of aerosol particulates.^17^

When comparing particle levels at the three distances within each evacuation scenario, *P* values were < 0.001 in all cases except one, indicating significant differences between distances for each evacuation method. The one exception, no evacuation, the post-hoc *P* value for comparing 0.9 and 1.8 meters was 0.865, leading to the acceptance of the null hypothesis that the aerosol levels between these distances are similar.

Finally, the four evacuation methods were compared against each other at each distance. In all cases except one, each evacuation method was significantly different from the others with a *P* value < 0.001. The exception occurred at 1.8 meters (6 feet), where no evacuation was matched with high-volume evacuation. In this comparison, post-hoc testing still revealed a significant difference with a *P* value of 0.005.

**Fig. 6:**
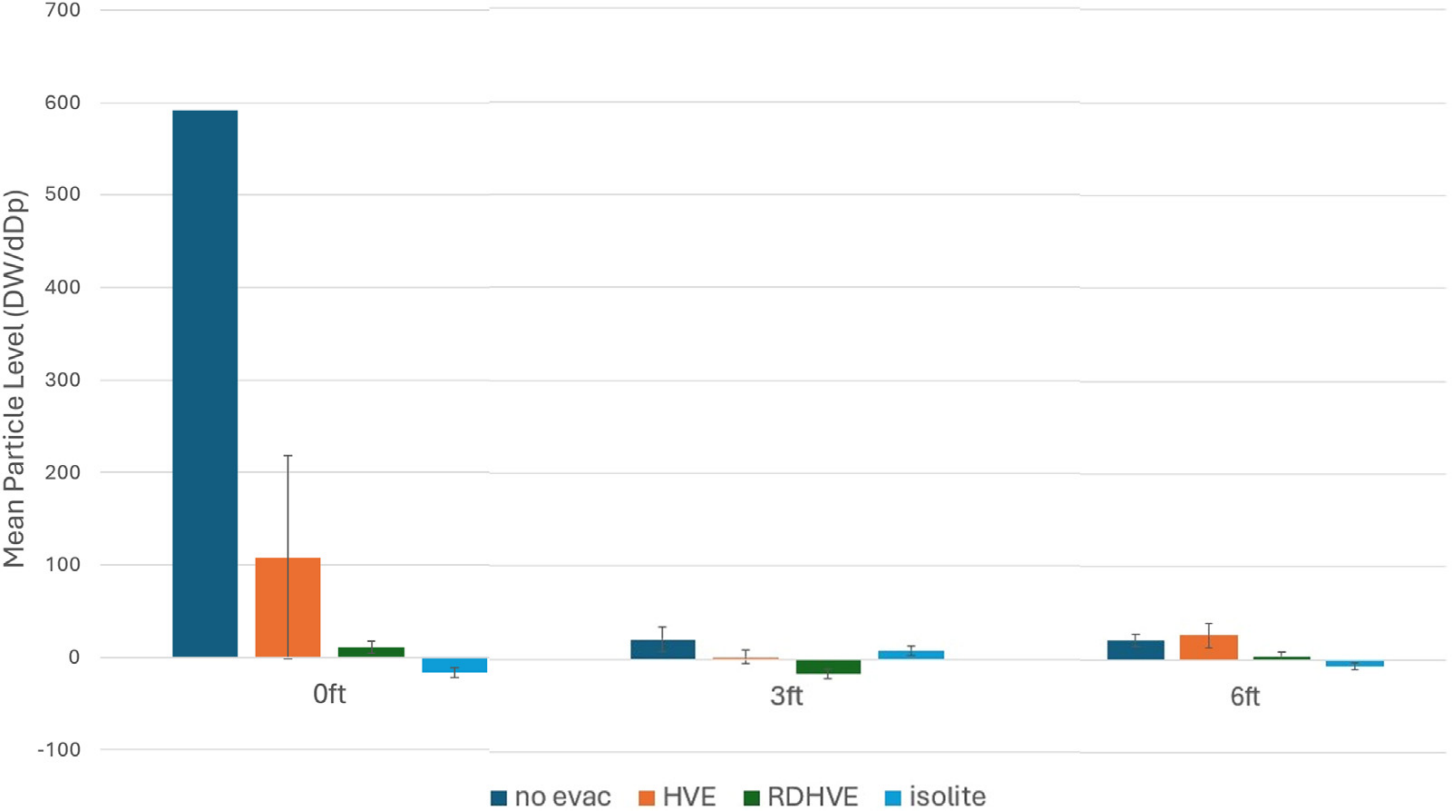
**Mean particle concentration at various distances with various methods of aerosol mitigation**. Mean concentrations of 0.337 µm particles, over a period of 7 minutes, were measured at various distances from the high-speed dental handpiece, using various methods of aerosol mitigation. Concentrations are presented in dW/dDp with s.d. ● = 0 m; ● = 0.9 m (3 ft); ● = 1.8 m (6 ft).

Isolite provided the largest mean drop from the control, with high-volume evacuation with a rubber dam at a close second place. While high-volume evacuation also showed a significant decrease in aerosols from no evacuation, the decrease was not as sharp as the other two evacuation methods (Figure 6).

## Discussion

The pattern of particle counts over time at 0 m distance differed from those at a distance of 0.9 and 1.8 m (3 feet and 6 feet, respectively). At 0 m distance, the count initially increased, then decreased steadily over 400 s. At 0.9 and 1.8 m, the surge was 10 to 25 times lower.

Changes in airflow in the immediate vicinity of the handpiece over time could be a plausible explanation for this phenomenon.^18^ A dental high-speed handpiece dispenses water and air.

Initially, the effect of this airflow may be highly localized, permitting a buildup of sprayed water to concentrate near the handpiece. Over time, the continuously sprayed air may generate a broader and stronger air current, progressively removing more particles that had accumulated close to the handpiece.^19^ Our study showed no significant difference in particle counts between 0.9 m (3 ft) and 1.8 m (6 ft). If this reflects the behaviour of aerosol spread in the clinical setting, then the ‘6-foot rule’ proposed by the Centers for Disease Control may not be pertinent for the dental setting; similar findings were reported by Rupf et al^20^ and Li et al^21^ for dental handpieces, and by Kumar et al^22^ for ultrasonic scalers. However, due to the design of our investigation, such conclusions cannot be drawn with confidence. The only aerosol source was the dental handpiece; however, patient respiration would also be an aerosol source in the clinic. In addition, occasionally, coughing can occur during dental procedures, such as when a patient chokes on water or dental materials. Also, room air quality and dynamics, such as the location and volume of room ventilation and filtration, can affect particle distribution and concentrations.

Evacuation of any type examined in our study, particularly at the locations where the clinician and assistant perform their duties, significantly reduces aerosol exposure, which is consistent with the findings of other studies.^20^, ^21^, ^22^ HVE alone is inferior to the combination of HVE, RD, or Isolite (or equivalent). While all three methods utilize the same HVE hose and vacuum unit, the RD and Isolite reduce the volume of space shared by the dental handpiece and the HVE, which may result in more rapid airflow and, consequently, more potent suction in the proximity of the dental handpiece. These findings imply that when a procedure and a patient permit using HVE, RD, or Isolite, these instruments should be utilized for the highest protection against aerosol contamination. For patients or procedures that prohibit their use simultaneously, HVE should be utilized.

Evacuation is only one part of a full aerosol infection-control system. Patient screenings and temperature measurements may also reduce risk for disease transmission. Masks are also protective. Feres et al^23^ found that N95 masks reduced aerosol exposure the most effectively, followed by surgical masks and cloth masks. In addition, the study found that using a face shield in conjunction with a mask significantly reduced aerosol exposure compared to using a mask alone.

Our research has limitations. It did not consider potential concentrations of pathogens, including bacteria and viruses, in the aerosol particles. These microorganisms are embedded in saliva, which is viscous and adheres to buccal tissues. The water ejected by the high-speed dental handpiece may or may not contain such microorganisms. Therefore, microorganisms in the oral cavity, and water in a dental handpiece, may become airborne at different rates. Low levels of aerosolized oral microorganisms were detected during dental aerosol-generating procedures in a clinical study by Choudhary et al.^24^ In addition, the dental handpiece was run continuously for 600 seconds to ensure uniformity across all parameters, whereas in a clinical context, the handpiece would typically run for shorter durations. Shorter intervals with interspersed pauses could reduce aerosol concentrations. Also, the typical back-and-forth and turning movements of a dental handpiece will affect the flow of particles, and those movements were not replicated in this study. For this study, the handpiece remained in the posterior portion of the jaw; aerosol values may differ by location in the jaw. Finally, this study measured only the physical aerosol distribution and therefore does not necessarily directly translate to the risk of an infectious agent being dispersed. Although we collected baseline particle counts before each experiment, different rooms will have different airflows and filtration qualities, and those factors can have significant effects on particle distribution.^18^^,^^25^ Such details were limited in this study. Furthermore, we did not measure the airflow rate of the dental suction. Future studies should include the effect of HEPA filtration and specific room airflow dynamic data.

In conclusion, while a 0.9 m (3 ft) distance from a dental handpiece may reduce the amount of exposure to aerosols, a greater distance may not afford greater reduction. If additional studies continue to have similar findings, public policies regarding safe distances may be reconsidered. Nonetheless, any form of HVE is highly recommended and ideally should include an RD or Isolite. Future studies will evaluate the usefulness of other protection measures, as well as their effectiveness with patients in a clinical setting.

## Author contributions

Mustafa Radif and Shika Gupta: Conception of project, performance of experiments, writing of manuscript. Andrew Young and Eris Salmon: Data analysis, writing of manuscript. David Ojcius: Conception of project, writing of manuscript.

## Data availability

The data that support the findings of this study are available from the corresponding author upon reasonable request.

## Conflict of interest

The authors declare that they have no competing interests with this publication.
